# Derivation and external validation of a prediction model for pneumococcal urinary antigen test positivity in patients with community-acquired pneumonia

**DOI:** 10.1017/ash.2023.421

**Published:** 2023-10-06

**Authors:** Priscilla Kim, Michael B. Rothberg, Amy S. Nowacki, Pei-Chun Yu, David Gugliotti, Abhishek Deshpande

**Affiliations:** 1 Cleveland Clinic Lerner College of Medicine of Case Western Reserve University, Cleveland, OH, USA; 2 Center for Value-Based Care Research, Primary Care Institute, Cleveland Clinic, Cleveland, OH, USA; 3 Department of Quantitative Health Sciences, Cleveland Clinic, Cleveland, OH, USA; 4 Vaccine and Infectious Disease and Public Health Sciences Divisions, Fred Hutch Cancer Research Center, Seattle, WA, USA; 5 Department of Hospital Medicine, Cleveland Clinic, Cleveland, OH, USA; 6 Department of Infectious Diseases, Cleveland Clinic, Cleveland, OH, USA

## Abstract

**Objective:**

Derive and externally validate a prediction model for pneumococcal urinary antigen test (pUAT) positivity.

**Methods:**

Retrospective cohort study of adults admitted with community-acquired pneumonia (CAP) to 177 U.S. hospitals in the Premier Database (derivation and internal validation samples) or 12 Cleveland Clinic hospitals (external validation sample). We utilized multivariable logistic regression to predict pUAT positivity in the derivation dataset, followed by model performance evaluation in both validation datasets. Potential predictors included demographics, comorbidities, clinical findings, and markers of disease severity.

**Results:**

Of 198,130 Premier patients admitted with CAP, 27,970 (14.1%) underwent pUAT; 1962 (7.0%) tested positive. The strongest predictors of pUAT positivity were history of pneumococcal infection in the previous year (OR 6.99, 95% CI 4.27–11.46), severe CAP on admission (OR 1.76, 95% CI 1.56–1.98), substance abuse (OR 1.57, 95% CI 1.27–1.93), smoking (OR 1.23, 95% CI 1.09–1.39), and hyponatremia (OR 1.35, 95% CI 1.17–1.55). Negative predictors included IV antibiotic use in past year (OR 0.65, 95% CI 0.52–0.82), congestive heart failure (OR 0.72, 95% CI 0.63–0.83), obesity (OR 0.71, 95% CI 0.60–0.85), and admission from skilled nursing facility (OR 0.60, 95% CI 0.45–0.78). Model c-statistics were 0.60 and 0.67 in the internal and external validation cohorts, respectively. Compared to guideline-recommended testing of severe CAP patients, our model would have detected 23% more cases with 5% fewer tests.

**Conclusion:**

Readily available data can identify patients most likely to have a positive pUAT. Our model could be incorporated into automated clinical decision support to improve test efficiency and antimicrobial stewardship.

## Introduction

*Streptococcus pneumoniae* remains the most commonly identified bacterial cause of community-acquired pneumonia (CAP), the leading infectious cause of hospitalization and death in both children and adults.^1^ The pneumococcal urinary antigen test (pUAT), which detects the C-polysaccharide antigen common to all serotypes of *S. pneumoniae*, has emerged as an important diagnostic method for pneumococcal pneumonia.^2^ Compared to traditional microbiological methods like blood or sputum cultures, which are affected by inadequate sample collection, delayed processing, and prior antimicrobial therapy, pUATs have increased the rate of etiologic diagnosis for CAP by 11%–23%.^1–4^ Identification of *S. pneumoniae* as the causative pathogen is important, as doing so enables early targeted treatment and improved antimicrobial stewardship.^5^

It remains unclear, however, which patients should undergo pUAT. Pneumococcal urinary antigen test utilization varies widely among hospitals across the U.S. One large retrospective cohort study of adults admitted with CAP to 170 U.S. hospitals showed that hospital rates of UAT utilization ranged from 0% to 69%, underscoring the lack of consensus on when this test should be ordered.^6^ The 2019 American Thoracic Society/Infectious Diseases Society of America (ATS/IDSA) guidelines argue against routine pUAT because small randomized trials have failed to demonstrate improved outcomes following pUAT and targeted therapy.^7,8^ However, because other large observational studies^9,10^ have reported reduced mortality for patients receiving pUAT, especially among severe and very severe CAP cases, the 2019 ATS/IDSA guidelines make a weak recommendation to perform pUAT only in patients with severe CAP. A recent prospective study of 1941 patients, however, showed that only 4% of patients who met ATS/IDSA indications for pUAT had a positive result.^11^ This suggests that current guidelines for pUAT utilization identify a population with low diagnostic yield, resulting in unnecessary tests and wasted resources.

The optimal patient population for pUAT thus remains unknown. Stronger predictors of pUAT positivity must be identified to improve test efficiency and identify opportunities for improved antimicrobial stewardship. Previous attempts to develop prediction models for pUAT positivity have been limited by small sample sizes, absent multivariable analyses, and the inclusion of variables that are not easily extracted from the electronic medical record (EMR).^11–16^ Importantly, to date, no prediction model has been validated. The objective of our study was to derive and externally validate a risk model to predict pUAT positivity in adults hospitalized with CAP and compare the efficiency of our model to that of guideline-recommended testing.

## Methods

This study was approved by Cleveland Clinic Institutional Review Board (18-1237, 15-1254, 16-1035). All analyses were conducted in R version 4.1.0. The Transparent Reporting of a multivariable prediction model for Individual Prognosis Or Diagnosis guidelines were followed.^17^

### Study population

We conducted a retrospective cohort study of adults (aged ≥18 years) admitted with pneumonia to one of 177 U.S. hospitals in the Premier Healthcare Database from January 2010 to December 2015, or to a non-Premier hospital in the Cleveland Clinic Health System (CCHS) from January 2010 to December 2019. The Premier Healthcare Database is a large U.S. hospital discharge database that has been widely used for research and includes hospitals located in all regions of the U.S.^18^ It contains data elements including sociodemographic information, discharge *International Classification of Disease, Ninth Revision, Clinical Modification* (ICD-9-CM) diagnosis and procedure codes, hospital and physician information, and a date-stamped record of all items billed during hospitalization.

We included patients with a primary diagnosis of pneumonia (ICD-9-CM codes 480–488, 507.0) and a present-on-admission flag, or a principal diagnosis of respiratory failure (ICD-9-CM 518.81, 518.82, 518.84, 779.1) or sepsis (ICD-9-CM 785.52, 790.7, 995.91, 995.92, 038.x), paired with a secondary diagnosis of pneumonia present-on-admission. To increase our certainty of capturing patients with suspected CAP at the time of admission, we restricted our study population to patients who had chest imaging by hospital day 1 and at least three consecutive days of antimicrobial treatment. Patients were excluded if they had a diagnosis of cystic fibrosis or tuberculosis, or if they were hospitalized for less than 24 hours. If patients were hospitalized more than once, the most recent hospitalization was selected for inclusion.

### Data extraction

For each hospitalization, the following data were extracted: patient age, sex, race, principal and secondary diagnoses, sociodemographic information, medical comorbidities, antibiotics prior to admission, treatments during admission, and microbiological data. Variables for patients in the Premier database were identified from hospital discharge records and physician claims using ICD-9-CM and daily charge codes, while those for patients in the CCHS database also included ICD-10-CM codes and clinical and laboratory data. A list of ICD-9-CM and charge codes that were used during data extraction is included in the Supplementary Information.

### Derivation and validation cohorts

**Figure 1.**
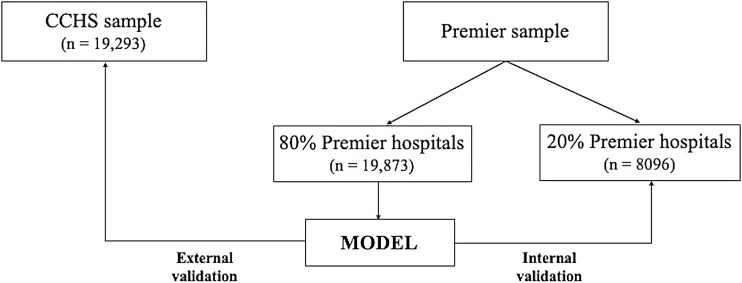
Schematic representation of internal and external validation employed.

To derive a prediction model for pUAT positivity, we first identified patients with CAP in the Premier Healthcare Database who underwent pUAT. We *a priori* decided to split the Premier sample by hospital, rather than at the patient level. We randomly selected 80% of the Premier hospitals to serve as the derivation cohort and used the remaining 20% as the internal validation cohort. A prediction model was then developed using the derivation cohort dataset (Figure 1). We applied the same inclusion and exclusion criteria to create an external validation cohort of patients admitted to one of 12 hospitals in the Cleveland Clinic Health System and who underwent pUAT (Figure 1). Only a small fraction (<1%) of patients were excluded from analyses for missing candidate predictors.

### Candidate predictor variables

Potential predictors were identified by reviewing the literature for risk factors for pneumococcal pneumonia that would be available at the time of admission. Candidate predictors included patient demographics, admission source, smoking status, clinical findings on admission (leukocytosis, hyponatremia, thrombocytopenia, confusion, and fever), medical comorbidities (e.g., chronic lung disease, congestive heart failure, chronic kidney disease, chronic liver disease, hypertension, diabetes, obesity, substance abuse, alcohol abuse, dementia, stroke history, and seizure/epilepsy), risk factors for multi-drug resistant organisms (residence in a skilled nursing facility (SNF), immunosuppression, dialysis, admission in the previous 6 months), hospital characteristics (location and teaching status), history of pneumococcal infection in the previous year, history of IV antibiotic use in the previous year, and pneumonia severity on admission (severe vs. non-severe). Severe CAP was defined as the need for vasopressors, invasive mechanical ventilation, or intensive care unit (ICU) admission in the first 48 h of hospitalization; this definition utilized the major criteria for severe CAP listed in the 2007 ATS/IDSA guidelines.^19^ History of pneumococcal infection in the previous year was defined as any positive test for *S. pneumoniae* (UAT, blood culture, sputum culture, or pleural fluid culture) in the previous 1 year prior to the admission date. Additional details on variable definitions used in the CCHS dataset are included in the Supplementary Information.

### Statistical methods

In the derivation cohort, all candidate predictor variables were included as potential covariates in a full multivariable logistic regression model. The primary outcome was pUAT result. Several variable selection methods were explored to obtain a parsimonious model: a) stepwise backward elimination with a significance level of 0.005 for variable retention, b) backward elimination by Akaike information criterion (AIC), and c) random forest. The model that represented the best trade-off between number of variables and predictive power was then selected as the final model and applied to both the internal and external validation cohorts. Model discrimination was compared based on the C-statistic. Model calibration was evaluated graphically by grouped bar charts of observed vs. predicted proportions of positive pUATs within increasing deciles of predicted probability. In the external validation cohort, a multiplicative scalar that minimized the total error between observed and predicted probabilities was determined and applied (Supplementary Information).

To evaluate the clinical benefit of our prediction model, we compared the efficiency of our model to that of the 2019 ATS/IDSA guidelines, which recommend pUAT only in patients with severe CAP. To do this, we used the pUAT positivity rate for severe CAP patients in each sample as the predicted probability threshold. Patients with a predicted probability greater than this cut-off value would have been tested using our model. We then compared the number of tests that would have been performed and the number of cases that would have been detected using our model, as compared to testing only patients with severe CAP.

## Results

The Premier dataset consisted of 198,130 patients admitted with CAP, while the CCHS dataset consisted of 115,404 patients admitted with CAP. Of 198,130 Premier patients admitted with CAP, 27,970 (14.1%) underwent pUAT, of whom 1962 (7.0%) tested positive. Characteristics of Premier patients with and without pUAT are shown in Supplemental Table 1. Overall, there were very small differences in patient-level characteristics between those who did and did not undergo pUAT. Patients with pUAT were not more likely to have severe CAP on admission, compared to those who did not undergo pUAT (22% vs. 23%).

### Characteristics of the cohorts

Characteristics of patients in the derivation, internal validation, and external validation cohorts are shown in Table 1. The median age of patients ranged from 69 to 71 years. All three cohorts had similar proportions of patients with hypertension (65%–71%) and type 2 diabetes (32%–35%). Compared with Premier patients, CCHS patients were more likely to have multiple comorbidities and severe CAP on admission (40% vs. ∼23%). The rate of pUAT positivity was lowest in the CCHS external validation cohort (2.0%), compared to the derivation (7.4%) and internal validation (6.2%) cohorts.

**Table 1.** Baseline characteristics of patients in the derivation, internal validation, and external validation cohorts

Characteristic	Cohort		
	Derivation, *N* = 19,873^1^	Internal validation, *N* = 8,096^1^	External validation, *N* = 19,293^1^
Age	69 (55, 80)	71 (57, 82)	69 (57, 80)
Female sex	9,978 (50%)	4,153 (51%)	9,606 (50%)
Race			
* Black*	3,040 (15%)	1,225 (15%)	3,287 (17%)
* White*	15,537 (78%)	6,405 (79%)	14,756 (76%)
* Other*	1,254 (6.3%)	463 (5.7%)	853 (4.4%)
* Unknown*	42 (0.2%)	3 (<0.1%)	397 (2.1%)
Smoker	4,707 (24%)	1,654 (20%)	3,700 (19%)
Alcohol abuse	1,044 (5.3%)	308 (3.8%)	1,966 (10%)
Substance abuse	934 (4.7%)	270 (3.3%)	1,875 (9.7%)
Obesity	3,170 (16%)	1,166 (14%)	7,083 (37%)
Chronic lung disease	9,622 (48%)	4,051 (50%)	11,767 (61%)
Congestive heart failure	5,380 (27%)	2,391 (30%)	7,737 (40%)
Chronic liver disease	753 (3.8%)	246 (3.0%)	1,005 (5.2%)
Chronic kidney disease	3,501 (18%)	1,401 (17%)	5,219 (27%)
Valvular disease	2,007 (10%)	528 (6.5%)	2,819 (15%)
Pulmonary circulation disease	1,811 (9.1%)	621 (7.7%)	1,769 (9.2%)
Peripheral vascular disease	1,662 (8.4%)	531 (6.6%)	5,052 (26%)
Hypertension	12,829 (65%)	5,285 (65%)	13,609 (71%)
Diabetes	6,365 (32%)	2,577 (32%)	6,786 (35%)
Dementia	1,822 (9.2%)	836 (10%)	1,890 (9.8%)
Metastatic cancer	751 (3.8%)	299 (3.7%)	2,179 (11%)
Solid tumor without metastasis	739 (3.7%)	319 (3.9%)	4,493 (23%)
History of stroke/cerebrovascular disease	1,811 (9.1%)	673 (8.3%)	4,555 (24%)
Seizure/epilepsy	914 (4.6%)	378 (4.7%)	1,426 (7.4%)
Low functional status	3,025 (15%)	1,587 (20%)	2,757 (14%)
Immunosuppressed	3,047 (15%)	1,077 (13%)	3,684 (19%)
Admit from SNF	1,274 (6.4%)	886 (11%)	582 (3.0%)
Admission in past 1 year	2,240 (11%)	997 (12%)	9,319 (48%)
Admission in past 6 months	1,527 (7.7%)	696 (8.6%)	7,709 (40%)
Leukocytosis	1,342 (6.8%)	430 (5.3%)	11,644 (60%)
Thrombocytopenia	1,372 (6.9%)	463 (5.7%)	5,122 (27%)
Hyponatremia	3,010 (15%)	791 (9.8%)	2,148 (11%)
Confusion	376 (1.9%)	149 (1.8%)	1,749 (9.1%)
Fever	820 (4.1%)	256 (3.2%)	5,754 (30%)
Urban/Rural			
* Rural*	875 (4.4%)	936 (12%)	0 (0%)
* Urban*	18,998 (96%)	7,160 (88%)	19,293 (100%)
Teaching hospital	10,770 (54%)	4,346 (54%)	12,481 (65%)
Region			
* Midwest*	5,100 (26%)	2,175 (27%)	18,798 (97%)
* Northeast*	2,025 (10%)	2,785 (34%)	0 (0%)
* South*	11,168 (56%)	2,954 (36%)	495 (2.6%)
* West*	1,580 (8.0%)	182 (2.2%)	0 (0%)
IV antibiotics in past 3 months	794 (4.0%)	353 (4.4%)	6,595 (34%)
IV antibiotics in past 1 year	1,795 (9.0%)	787 (9.7%)	8,000 (41%)
SP infection in past 1 year	93 (0.5%)	49 (0.6%)	115 (0.6%)
Severe CAP on admission	4,355 (22%)	1,910 (24%)	7,648 (40%)
Positive pUAT	1,462 (7.4%)	500 (6.2%)	394 (2.0%)

Abbreviations: SNF, skilled nursing facility; SP, *Streptococcus pneumoniae.*

^1^Median (IQR) for continuous; *n* (%) for categorical.

### Characteristics of patients with positive vs. negative pUAT in the derivation cohort

Several variables were significantly associated with a positive pUAT in univariate analyses. Table 2 shows characteristics of patients with positive and negative pUATs in the derivation cohort. Compared to patients with negative pUATs, those with positive tests were younger (median age 65 vs. 69 years), were current smokers (31% vs. 23%), had substance abuse (8.6% vs. 4.4%), had severe CAP at the time of admission (31% vs. 21%), had hyponatremia at the time of admission (20% vs. 15%), and had a history of pneumococcal infection in the previous 1 year (1.8% vs. 0.4%). They were less likely to have obesity (12% vs. 16%) and congestive heart failure (20% vs. 28%).

**Table 2.** Characteristics of Premier hospital patients with positive vs. negative pneumococcal urinary antigen tests within the derivation cohort

	Positive pUAT, *N* = 1462 (7.4%)^1^	Negative pUAT, *N* = 18411 (93%)^1^	*p*-value^2^
Age	65 (55, 78)	69 (56, 80)	**<0.001**
Female sex	772 (53%)	9,206 (50%)	**0.039**
Race			
* Black*	254 (17%)	2,786 (15%)	
* White*	1,097 (75%)	14,440 (78%)	
* Other*	109 (7.5%)	1,145 (6.2%)	
* Unknown*	2 (0.1%)	40 (0.2%)	
Smoker	451 (31%)	4,256 (23%)	**<0.001**
Alcohol abuse	126 (8.6%)	918 (5.0%)	**<0.001**
Substance abuse	126 (8.6%)	808 (4.4%)	**<0.001**
Obesity	170 (12%)	3,000 (16%)	**<0.001**
Chronic lung disease	734 (50%)	8,888 (48%)	0.2
Congestive heart failure	295 (20%)	5,085 (28%)	**<0.001**
Chronic liver disease	83 (5.7%)	670 (3.6%)	**<0.001**
Chronic kidney disease	233 (16%)	3,268 (18%)	0.080
Valvular disease	113 (7.7%)	1,894 (10%)	**0.002**
Pulmonary circulation disease	104 (7.1%)	1,707 (9.3%)	**0.006**
Peripheral vascular disease	90 (6.2%)	1,572 (8.5%)	**0.002**
Hypertension	838 (57%)	11,991 (65%)	**<0.001**
Diabetes	380 (26%)	5,985 (33%)	**<0.001**
Dementia	112 (7.7%)	1,710 (9.3%)	**0.038**
Deficiency anemia	399 (27%)	5,401 (29%)	0.10
Pressure ulcer	65 (4.4%)	905 (4.9%)	0.4
Stroke/cerebrovascular disease	101 (6.9%)	1,710 (9.3%)	**0.002**
Seizure/epilepsy	66 (4.5%)	848 (4.6%)	0.9
Low functional status	188 (13%)	2,837 (15%)	**0.009**
Immunosuppressed	217 (15%)	2,830 (15%)	0.6
Admit from SNF	58 (4.0%)	1,216 (6.6%)	**<0.001**
Admission in past 1 year	136 (9.3%)	2,104 (11%)	**0.013**
Admission in past 6 months	85 (5.8%)	1,442 (7.8%)	**0.005**
Leukocytosis	88 (6.0%)	1,254 (6.8%)	0.2
Thrombocytopenia	129 (8.8%)	1,243 (6.8%)	**0.003**
Hyponatremia	289 (20%)	2,721 (15%)	**<0.001**
Confusion	32 (2.2%)	344 (1.9%)	0.4
Fever	51 (3.5%)	769 (4.2%)	0.2
Urban/Rural			0.8
* Rural*	66 (4.5%)	809 (4.4%)	
* Urban*	1,396 (95%)	17,602 (96%)	
Teaching hospital	759 (52%)	10,011 (54%)	0.069
Region			0.086
* Midwest*	338 (23%)	4,762 (26%)	
* Northeast*	162 (11%)	1,863 (10%)	
* South*	851 (58%)	10,317 (56%)	
* West*	111 (7.6%)	1,469 (8.0%)	
IV antibiotics in past 3 months	40 (2.7%)	754 (4.1%)	**0.011**
IV antibiotics in past 1 year	102 (7.0%)	1,693 (9.2%)	**0.004**
SP infection in past 1 year	27 (1.8%)	66 (0.4%)	**<0.001**
Severe CAP on admission	447 (31%)	3,908 (21%)	**<0.001**

Abbreviations: SNF, skilled nursing facility; SP, *Streptococcus pneumoniae.*

^1^Median (IQR) for continuous; *n* (%) for categorical.

^2^Wilcoxon rank sum test; Pearson’s Chi-squared test; Fisher’s exact test.

### Predictors of pneumococcal UAT positivity in the derivation cohort

Our final model was developed from stepwise backward elimination with a significance level of 0.005 for variable retention and included 14 variables: race, admission from an SNF, congestive heart failure, hypertension, obesity, substance abuse, diabetes, smoking, hyponatremia, teaching hospital, region, IV antibiotic use in the past year, severe CAP on admission, and history of pneumococcal infection in the past year. Odds ratios (ORs) and 95% confidence intervals (CIs) for predictors are shown in Table 3. History of pneumococcal infection in the past year (OR 6.99, 95% CI 4.27–11.46), severe CAP on admission (OR 1.76, 95% CI 1.56–1.98), substance abuse (OR 1.57, 95% CI 1.27–1.93), smoking (OR 1.23, 95% CI 1.09–1.39), and hyponatremia (OR 1.35, 95% CI 1.17–1.55) were most strongly associated with pUAT positivity. IV antibiotic use in the past year (OR 0.65, 95% CI 0.52–0.82), congestive heart failure (OR 0.72, 95% CI 0.63–0.83), obesity (OR 0.71, 95% CI 0.60–0.85), and admission from a skilled nursing facility (OR 0.60, 95% CI 0.45–0.78) were negative predictors of pUAT positivity. Results from alternative variable selection methods (backward elimination by AIC, random forest) are presented in Supplementary Table 2.

**Table 3.** Associations between patient/hospital characteristics and pUAT positivity using multivariable logistic regression in the derivation cohort

Characteristic	Adjusted OR	95% CI	*P*-value
Race (ref = Black)			<0.001
* *White	0.79	0.68–0.92	
* *Other	1.14	0.88–1.47	
* *Unknown	0.74	0.18–3.11	
Admit from SNF	0.60	0.45–0.78	<0.001
Congestive heart failure	0.72	0.63–0.83	<0.001
Hypertension	0.85	0.76–0.95	0.005
Obesity	0.71	0.60–0.85	<0.001
Substance abuse	1.57	1.27–1.93	<0.001
Diabetes	0.83	0.73–0.94	0.004
Smoker	1.23	1.09–1.39	0.001
Hyponatremia	1.35	1.17–1.55	<0.001
Teaching hospital	0.85	0.76–0.95	0.004
Region (ref = Midwest)			0.001
* *Northeast	1.30	1.06–1.58	
* *South	1.20	1.05–1.37	
* *West	0.88	0.69–1.12	
IV antibiotics in past year	0.65	0.52–0.82	<0.001
Severe CAP on admission	1.76	1.56–1.98	<0.001
SP infection in past year	6.99	4.27–11.46	<0.001

Abbreviations: SNF, skilled nursing facility; SP, *Streptococcus pneumoniae.*

Model intercept = −2.354.

### Prediction model performance

**Figure 2.**
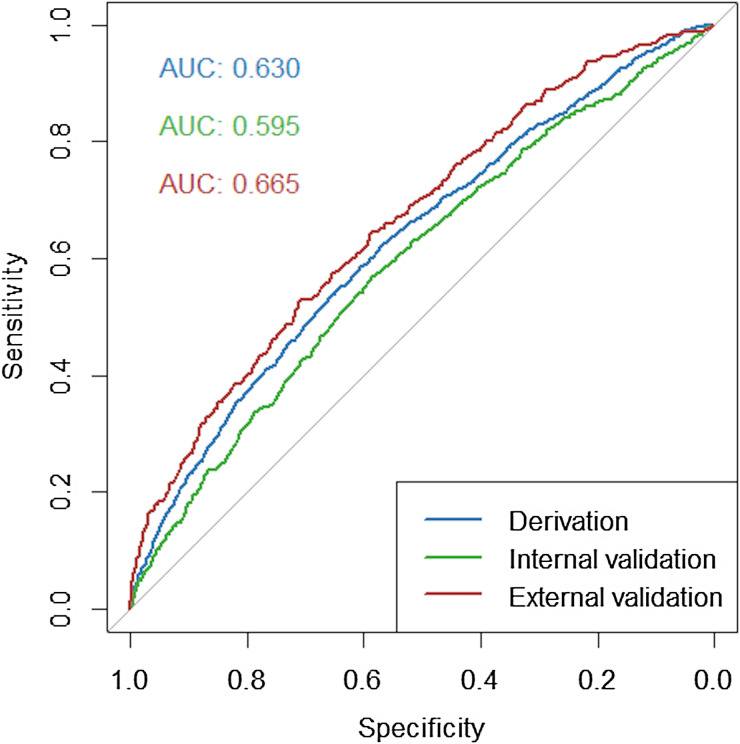
Receiver operating characteristic (ROC) curves for derivation, internal validation, and Cleveland Clinic Health Systems (CCHS) external validation samples.

**Figure 3.**
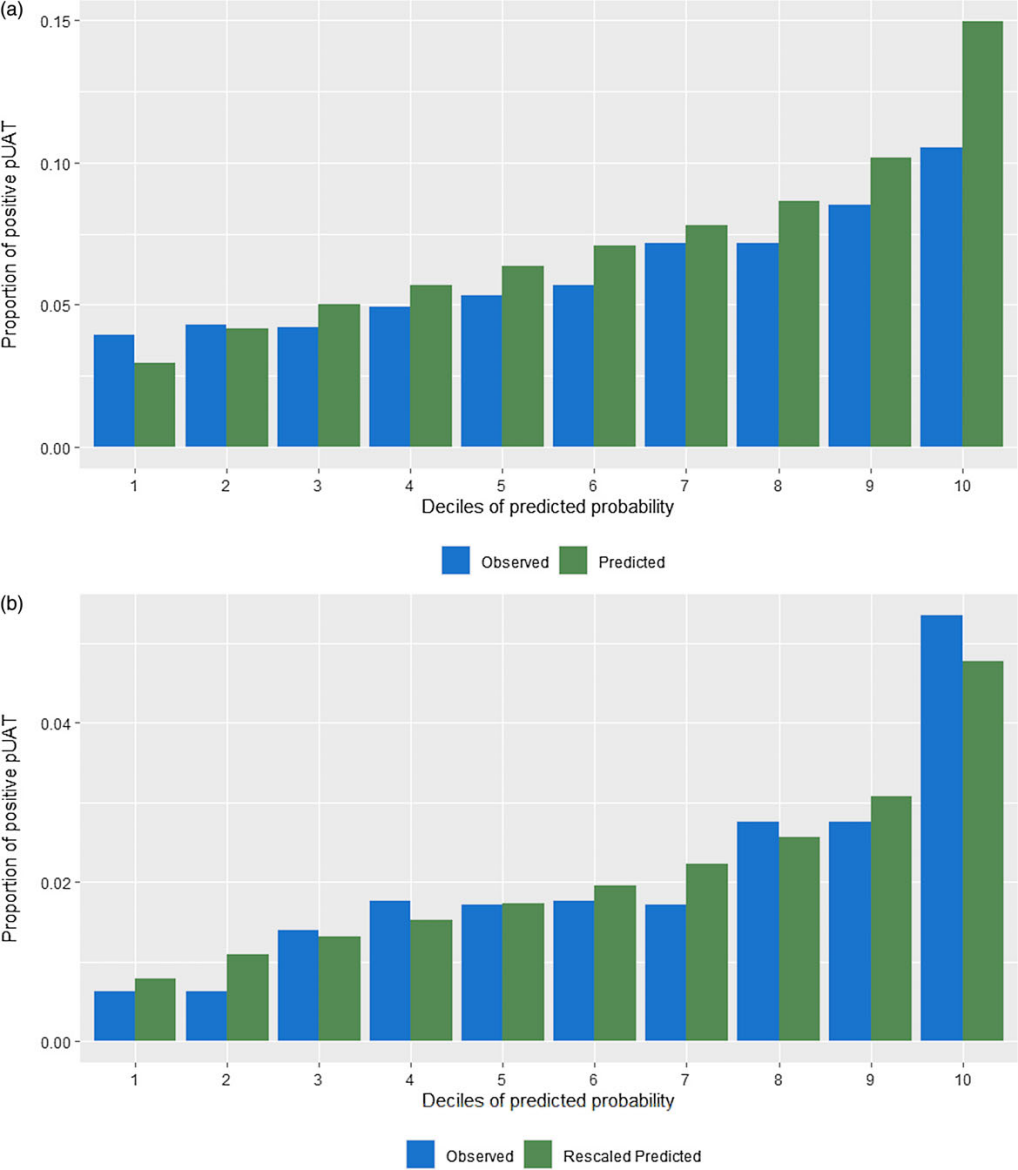
Observed vs. predicted pUAT positivity proportions in the Premier internal validation and CCHS external validation samples, based on a 14-variable prediction model derived from the Premier 80% derivation sample. (A) Premier 20% hold-out internal validation sample, (B) CCHS external validation sample. Rescaled calibration plot where a multiplicative scalar of 0.36 was applied to the original predicted probabilities.

Receiver operating characteristic curves for our model in the derivation, internal validation, and external validation cohorts are superimposed in Figure 2. Our model had a C-statistic of 0.63 in the derivation sample, 0.60 in the internal validation sample, and 0.67 in the external CCHS sample. Figure 3 shows the proportion of positive pUATs in the two validation samples by deciles of predicted risk. Our model demonstrated excellent calibration in the internal validation cohort, especially across the first nine deciles (Figure 3A). In the CCHS external validation sample, our model overestimated the risk of pUAT positivity (Supplemental Figure 1); a data-determined multiplicative scalar of 0.36 was applied to the original predicted probabilities to adjust the calibration (Figure 3B). Details of the re-scaling process are included in the Supplementary Information. In the Premier sample, of the 1962 patients who tested positive, 1114 (56.8%) were in the top four risk deciles. In the CCHS sample, of the 394 patients with positive pUATs, 242 (61.4%) were in the top four risk deciles.

Of the 27,970 patients in the Premier sample who underwent pUAT, 6265 (22.4%) were guideline-concordant and had severe CAP on admission, of whom 575 (9.2%) tested positive. At a threshold of predicted risk of 9.2%, 5971 patients would have been tested using our prediction model, of whom 707 (11.8%) would have tested positive. Compared to testing only patients with severe CAP, our model would have detected 23% more cases with 5% fewer tests in the Premier dataset. In the external validation cohort, our model would have detected a greater proportion of pneumococcal cases (3.8% vs. 2.6%, *p* < 0.001) with 37.6% fewer tests, when compared to testing only patients with severe CAP. Additional details are provided in the Supplemental Information.

## Discussion

In this large retrospective cohort study, we derived and externally validated a prediction model for pUAT positivity in patients admitted with CAP. We derived a model that balanced clinical utility with predictive power and identified key risk factors for pUAT positivity, including history of pneumococcal infection in the past year, severe CAP on admission, substance abuse, smoking, and hyponatremia. Negative predictors included IV antibiotic use in the past year, congestive heart failure, and obesity. Importantly, because these factors can be easily extracted from the EMR, our model can be incorporated into automated clinical decision support. Compared to guideline-recommended testing, our model detected more cases of pneumococcal pneumonia with fewer tests.

Several studies have reported similar predictors of pUAT positivity in CAP patients.^11–16^ One prospective study in Spain^12^ found that Pneumonia Severity Index (PSI) risk class ≥IV was a significant predictor of pUAT positivity, while another in Utah^16^ reported ICU admission as a significant risk factor. Smoking,^15^ hyponatremia,^12^ and female sex^12,14^ have also been reported to be independent predictors, while prior antibiotic treatment^12^ has been found to be a negative predictor.

To the best of our knowledge, only one other study has evaluated model performance.^12^ Their model contained nine variables: female sex, heart rate ≥125 beats per minute, systolic blood pressure <90 mmHg, oxygen saturation <90%, no prior antibiotic treatment, pleuritic chest pain, chills, pleural effusion, and blood urea nitrogen (BUN) >30 mg/dL. With a C-statistic of 0.64 (95% CI, 0.58–0.70), their model’s discrimination was similar to ours. However, unlike our model, theirs required several variables that would not be easily extracted from the EMR, limiting the feasibility of their model to be incorporated into automated clinical decision support.

Our model is the first to be externally validated. In the external validation cohort, our model offered fair discrimination, but it overestimated the risk of pUAT positivity, likely due to the large differences in pUAT positivity rates between the CCHS (2.0%) and Premier derivation cohort (7.4%). These differences can likely be explained by significant geographic, hospital, and temporal dissimilarities between the two datasets. The observed proportion of positive pUATs, however, increased with every increase in decile of predicted probability, suggesting that our predictions could be multiplied by a scalar to adequately adjust the calibration. After applying a data-determined multiplicative scalar that minimized the total error between observed and predicted probabilities, our model’s calibration was significantly improved.

This study has several limitations. First, because the Premier database is primarily an administrative dataset based on discharge ICD-9 codes, it is possible that we may have underestimated the presence of risk factors, and the generalizability of our model could be limited. Additionally, some risk factors (e.g., substance use disorder) are very broad terms without granularity. However, the Premier database includes detailed date-stamped billing records during hospitalization, increasing our confidence in the specificity of the diagnosis and procedure codes. Second, the Premier dataset lacks many clinical variables that could not be included in our model. However, we validated our model on a clinically rich dataset of CCHS patients. Third, due to limitations of the Premier database, we used surrogate markers of severity to define severe CAP, rather than utilizing both the major and minor criteria of the 2007 ATS/IDSA guidelines. Next, information on patients’ pneumococcal vaccination status was not available in our datasets. Finally, our model was derived using data only from patients who underwent pUAT, which is not routinely performed in all CAP patients and thus may limit the generalizability of our model.

Our study has important implications. Current ATS/IDSA guidelines recommend pUAT only in patients with severe CAP,^7^ but it appears that clinicians are not targeting this population when ordering pUATs. Among Premier patients, we found that those who underwent pUAT were not more likely to have severe CAP on admission, and there were very small differences in patient-level characteristics between those who were and were not tested. Even when guideline recommendations are followed, the pUAT costs approximately $425 per positive test result due to the low positive test prevalence.^11,20^ By detecting more cases of pneumococcal pneumonia with fewer tests, our model could significantly improve efficiency of testing, making the pUAT more cost-effective. More importantly, however, early identification of *S. pneumoniae* as the causative pathogen increases opportunities for antimicrobial stewardship by enabling early targeted treatment and antibiotic de-escalation. The next logical step would be to incorporate our prediction model into the EMR to provide point-of-care clinical decision support to providers. A well-designed randomized controlled trial could then determine whether use of our model can promote antibiotic de-escalation and improve outcomes.

In summary, we derived and externally validated a prediction model for pUAT positivity. Because our model contains variables that can be easily extracted from the EMR, it has the potential to be incorporated into the EMR to improve efficiency of testing, facilitate early diagnosis of pneumococcal pneumonia, and enable antibiotic de-escalation.

## Supporting information

Kim et al. supplementary material Kim et al. supplementary material

## Supplementary material

For supplementary material accompanying this paper visit http://doi.org/10.1017/ash.2023.421. click here to view supplementary material
