# Supplementary material for: Derivation and external validation of a prediction model for pneumococcal urinary antigen test positivity in patients with community-acquired pneumonia
Source: Antimicrob Steward Healthc Epidemiol. 2023 Oct 6;3(1):e166. doi: 10.1017/ash.2023.421 (PMC10644161; doi:10.1017/ash.2023.421)
Supplement: Kim et al. supplementary material [file S2732494X23004217sup001.docx]

**Supplemental information**

**ICD-9-CM or charge codes used to define variables in Premier dataset**

| **Variable** | **ICD-9-CM or charge codes** |
| --- | --- |
| Pneumonia | ICD-9-CM codes 480–488, 507.0 |
| Respiratory failure | ICD-9-CM 518.81, 518.82, 518.84, 779.1 |
| Sepsis | ICD-9-CM 785.52, 790.7, 995.91, 995.92, 038.x |
| Smoking | ICD-9-CM codes 305.1x  Charge codes '250250106140000', '25025010615000’, '250250106880000', '250250106890000', '250257046850000', '250257046880000', '250257046930000', '250257046940000', '250257046950000', '250257046960000', '250250046970000', '250250104870000', '250250104880000', '250250104890000',' 250999012480000', '250999021310000', '250257046860000', '250257046870000', '250257046890000', '250257046900000', ‘250257046910000', '250257046920000' on any hospital day |
| Alcohol abuse | ICD-9-CM codes 265.2, 291.1, 291.2, 291.3, 291.5, 291.8, 291.9, 303.0, 303.9, 305.0, 357.5, 425.5, 535.3, 571.0, 571.1, 571.2, 571.3, 980, V11.3 |
| Substance abuse | ICD-9-CM codes 292.0,292.82-292.89, 292.9, 304.0-304.4, 304.5-304.9, 305.2-305.9, 648.3 |
| Obesity | ICD-9-CM code 278.0 |
| Chronic lung disease | ICD-9-CM codes 490-496, 500-505, 506.4 |
| Congestive heart failure | ICD-9-CM codes 398.91, 402.01, 402.11, 402.91, 404.01, 404.03, 404.11, 404.13, 404.91, 404.93, 425.4, 425.5, 425.7-425.9, 428 |
| Chronic liver disease | ICD-9-CM codes 070.22, 070.23, 070.32, 070.33, 070.44, 070.54, 070.6, 070.9, 456.0, 456.1, 456.2, 570, 571, 572.2, 572.3, 572.4, 572.8, 573.3, 573.4, 573.9, 573.9, V42.7 |
| Chronic kidney disease | ICD-9-CM codes 585.1,585.2, 585.3, 585.4, 585.5, 585.9 |
| Hypertension | ICD-9-CM codes 401-405 |
| Diabetes | ICD-9-CM codes 250.0-250.9 |
| Dementia | ICD-9-CM codes 290, 291.1, 291.2, 292.82, 292.83, 294.0, 294.1, 294.8, 294.9,331.0, 331.1,331.7, 331.9, 797,331.2, 331.3, 331.4, 331.5, 331.82 |
| Leukocytosis | ICD-9-CM code 288.60 |
| Thrombocytopenia | ICD-9-CM codes 287.5, 287.30, 287.39 |
| Hyponatremia | ICD-9-CM code 276.1 |
| Confusion | ICD-9-CM code 780.97 |
| Fever | ICD-9-CM codes 780.60, 780.61, 780.62, 780.63 |

**Candidate predictor variables**

For Cleveland Clinic Health Systems (CCHS) patients, obesity was defined as having a BMI of >30. Immunosuppression was defined as having any of the following: AIDS, leukemia, lymphoma, Hodgkin’s disease, multiple myeloma, sickle cell disease, history of splenectomy, history of transplantation, ICD-9-CM codes 277.2, 277.6, or 279.x, or ICD-10-CM codes D80.0-D89.9, D71, Z94.x, Z92.25, Z92.21, or Z92.22. Leukocytosis was defined as having any white blood cell count of >11.0 k/uL (1). Hyponatremia was defined as having any serum sodium of <130 mmol/L (2). Thrombocytopenia was defined as having any platelet count of <150 k/uL (3). Fever was defined as having any temperature >100.4°F (38°C) (4).

**Determining calibration adjustment in the CCHS external validation sample**

In the CCHS external validation cohort, the observed proportion of positive pUATs increased with every increase in decile of predicted probability, suggesting that our predictions could be multiplied by a data-determined scalar to adjust the calibration. We sought to identify a multiplicative scalar that would minimize the total error between observed and rescaled predicted probabilities.

Below is an example of the process we used to calculate the total error between observed and rescaled predicted probabilities after applying a multiplicative scalar to the original predicted probabilities in the CCHS external validation dataset. In the below example, a multiplicative scalar of 0.36 was applied.

|  | **Decile mean** | | |  |
| --- | --- | --- | --- | --- |
| **Decile** | **Original predicted probability** | **Observed probability** | **Rescaled predicted probability^‡^**  **(original predicted probability * scalar)** | **Error (Rescaled predicted – observed probability)** |
| 1 | 0.021948 | 0.006218 | 0.007901 | 0.001684 |
| 2 | 0.030303 | 0.006218 | 0.010909 | 0.004691 |
| 3 | 0.036577 | 0.01399 | 0.013168 | 0.000822 |
| 4 | 0.04219 | 0.017626 | 0.015188 | 0.002437 |
| 5 | 0.048127 | 0.017107 | 0.017326 | 0.000219 |
| 6 | 0.05439 | 0.017626 | 0.019581 | 0.001955 |
| 7 | 0.06165 | 0.017107 | 0.022194 | 0.005087 |
| 8 | 0.071106 | 0.027475 | 0.025598 | 0.001877 |
| 9 | 0.085435 | 0.027475 | 0.030757 | 0.003281 |
| 10 | 0.132665 | 0.053396 | 0.04776 | 0.005636 |
|  | | | **Error sum** | **0.027689** |

**^‡^** In this example, scalar = 0.36

This process was repeated for various multiplicative scalars (see table below). We determined that a multiplicative scalar of 0.36 resulted in the minimum total error between observed and rescaled predicted probabilities. This scalar of 0.36 was thus selected for the re-scaled calibration plot.

| **Multiplicative scalar** | **Error sum** |
| --- | --- |
| 0.1 | 0.145798 |
| 0.2 | 0.087359 |
| 0.25 | 0.060855 |
| 0.3 | 0.038175 |
| 0.35 | 0.028021 |
| **0.36** | **0.027689 (minimum)** |
| 0.37 | 0.027882 |
| 0.38 | 0.028075 |
| 0.39 | 0.029331 |
| 0.4 | 0.031678 |
| 0.5 | 0.087959 |

**Comparing the efficiency of our model to that of guidelines in the external validation sample**

As described in the methods, we used the pUAT positivity rate for severe CAP patients in each sample as the predicted probability threshold. Of the 19,293 patients in the Cleveland Clinic Health Systems (CCHS) external validation cohort who underwent pUAT, 7648 (39.6%) were guideline-concordant and had severe CAP on admission, of whom 197 (2.6%) tested positive. At a threshold of predicted risk of 2.6%, 4771 patients would have been tested using our prediction model, of whom 179 (3.8%) would have tested positive. Thus, in the external validation cohort, our model would have detected a greater proportion of cases (3.8% vs 2.6%, p<0.001) with 37.6% fewer pneumococcal urinary antigen tests, compared to testing only patients with severe CAP. The below table summarizes the ability of our prediction model to identify positive pUATs in the Premier dataset and Cleveland Clinic Health Systems external validation sample.

|  | **Premier sample** | **External validation  (CCHS) sample** |
| --- | --- | --- |
| **Total patients** | N = 27,970 | N = 19,293 |
| **No. who would be tested according to guidelines (severe CAP)** | 6265 (22.4%) | 7648 (39.6%) |
| Positive pUAT | 575 (9.2%) | 197 (2.6%) |
| **No. who would be tested according to our model** | 5971 (21.3%)^a^ | 4771 (24.7%)^b^ |
| Positive pUAT | 707 (11.8%) | 179 (3.8%) |

^a^ Threshold of predicted risk of 9.2%

^b^ Threshold of predicted risk of 2.6%

**Figure titles and legends**

**Supplemental Figure 1. Calibration plot in the CCHS external validation sample before and after application of a multiplicative scalar (0.36)**

**Supplemental Table 1. Characteristics of Premier patients admitted with community-acquired pneumonia with and without pneumococcal urinary antigen testing (pUAT)**

|  | **Overall,**  **N = 198,130^1^** | **With pUAT,**  **N=27,970 (14%)^1^** | **Without pUAT,**  **N=170,160 (86%)^1^** | **p-value^2^** |
| --- | --- | --- | --- | --- |
| **Demographics** |  |  |  |  |
| Age, year | 72 (58, 83) | 69 (56, 81) | 72 (59, 83) | **<0.001** |
| Female sex | 101,183 (51%) | 14,131 (51%) | 87,052 (51%) | **0.048** |
| Race |  |  |  | **<0.001** |
| Black | 24,019 (12%) | 4,265 (15%) | 19,754 (12%) |  |
| White | 151,641 (77%) | 21,943 (78%) | 129,698 (76%) |  |
| Other | 22,290 (11%) | 1,717 (6.1%) | 20,573 (12%) |  |
| Unknown | 180 (<0.1%) | 45 (0.2%) | 135 (<0.1%) |  |
| Smoker | 35,965 (18%) | 6,361 (23%) | 29,604 (17%) | **<0.001** |
| Alcohol abuse | 8,247 (4.2%) | 1,352 (4.8%) | 6,895 (4.1%) | **<0.001** |
| Drug abuse | 6,827 (3.4%) | 1,204 (4.3%) | 5,623 (3.3%) | **<0.001** |
| Obesity | 28,000 (14%) | 4,337 (16%) | 23,663 (14%) | **<0.001** |
| **Comorbidities** |  |  |  |  |
| Chronic lung disease | 91,503 (46%) | 13,673 (49%) | 77,830 (46%) | **<0.001** |
| Congestive heart failure | 56,118 (28%) | 7,771 (28%) | 48,347 (28%) | **0.030** |
| Chronic liver disease | 6,958 (3.5%) | 999 (3.6%) | 5,959 (3.5%) | 0.60 |
| Chronic kidney disease | 36,139 (18%) | 4,902 (18%) | 31,237 (18%) | **<0.001** |
| Valvular disease | 18,911 (9.5%) | 2,536 (9.1%) | 16,375 (9.6%) | **0.003** |
| Pulmonary circulation   disease | 15,943 (8.0%) | 2,432 (8.7%) | 13,511 (7.9%) | **<0.001** |
| Peripheral vascular   disease | 16,926 (8.5%) | 2,193 (7.8%) | 14,733 (8.7%) | **<0.001** |
| Hypertension | 130,425 (66%) | 18,115 (65%) | 112,310 (66%) | **<0.001** |
| Diabetes | 65,504 (33%) | 8,943 (32%) | 56,561 (33%) | **<0.001** |
| Dementia | 26,044 (13%) | 2,658 (9.5%) | 23,386 (14%) | **<0.001** |
| Deficiency anemia | 56,845 (29%) | 7,758 (28%) | 49,087 (29%) | **<0.001** |
| Pressure ulcer | 11,774 (5.9%) | 1,387 (5.0%) | 10,387 (6.1%) | **<0.001** |
| Stroke/cerebrovascular   disease | 18,545 (9.4%) | 2,484 (8.9%) | 16,061 (9.4%) | **0.003** |
| Low functional status | 29,358 (15%) | 4,612 (16%) | 24,746 (15%) | **<0.001** |
| **Risk for drug-resistant organisms** |  |  |  |  |
| Immunosuppressed | 27,101 (14%) | 4,124 (15%) | 22,977 (14%) | **<0.001** |
| Admit from SNF | 15,273 (7.7%) | 2,160 (7.7%) | 13,113 (7.7%) | >0.90 |
| Admission in past 1 year | 27,755 (14%) | 3,237 (12%) | 24,518 (14%) | **<0.001** |
| Admission in past 6   months | 19,906 (10%) | 2,223 (7.9%) | 17,683 (10%) | **<0.001** |
| **Clinical findings** |  |  |  |  |
| Leukocytosis | 10,063 (5.1%) | 1,772 (6.3%) | 8,291 (4.9%) | **<0.001** |
| Thrombocytopenia | 12,554 (6.3%) | 1,835 (6.6%) | 10,719 (6.3%) | 0.10 |
| Hyponatremia | 24,640 (12%) | 3,802 (14%) | 20,838 (12%) | **<0.001** |
| Confusion | 3,962 (2.0%) | 525 (1.9%) | 3,437 (2.0%) | 0.11 |
| Fever | 11,232 (5.7%) | 1,077 (3.9%) | 10,155 (6.0%) | **<0.001** |
| **Hospital characteristics** |  |  |  |  |
| Urban/Rural |  |  |  | **<0.001** |
| Rural | 24,159 (12%) | 1,811 (6.5%) | 22,348 (13%) |  |
| Urban | 173,971 (88%) | 26,159 (94%) | 147,812 (87%) |  |
| Teaching hospital | 80,134 (40%) | 15,116 (54%) | 65,018 (38%) | **<0.001** |
| Region |  |  |  | **<0.001** |
| Midwest | 48,620 (25%) | 7,275 (26%) | 41,345 (24%) |  |
| Northeast | 35,180 (18%) | 4,810 (17%) | 30,370 (18%) |  |
| South | 86,019 (43%) | 14,123 (50%) | 71,896 (42%) |  |
| West | 28,311 (14%) | 1,762 (6.3%) | 26,549 (16%) |  |
| **Past medical history** |  |  |  |  |
| IV antibiotics in past   3 months | 10,845 (5.5%) | 1,147 (4.1%) | 9,698 (5.7%) | **<0.001** |
| IV antibiotics in past   1 year | 22,647 (11%) | 2,582 (9.2%) | 20,065 (12%) | **<0.001** |
| SP infection in past 1   year | 531 (0.3%) | 142 (0.5%) | 389 (0.2%) | **<0.001** |
| **Severity of illness** |  |  |  |  |
| Severe CAP on   admission | 45,383 (23%) | 6,265 (22%) | 39,118 (23%) | **0.03** |

Abbreviations: SNF, skilled nursing facility; SP, *Streptococcus pneumoniae*

^1^median (IQR) for continuous; n(%) for categorical

^2^Wilcoxon rank sum test; Pearson’s Chi-squared test

**Supplemental Table 2: Associations between patient/hospital characteristics and pUAT positivity using multivariable logistic regression with different variable selection methods**

|  | **Backward elimination by AIC** | | | **Backward elimination (significance level 0.005)** | | | **Random forest** | | |
| --- | --- | --- | --- | --- | --- | --- | --- | --- | --- |
| **No. predictors** | 8 | | | 14 | | | 30 | | |
| **C-statistic** | 0.612 | | | 0.630 | | | 0.626 | | |
| **Model intercept** | -2.653 | | | -2.354 | | | -2.623 | | |
| **Characteristic** | **Adj OR** | **95% CI** | **P-value** | **Adj OR** | **95% CI** | **P-value** | **Adj OR** | **95% CI** | **P-value** |
| Age |  |  |  |  |  |  | 1.00 | 1.00-1.007 | 0.09 |
| Female sex |  |  |  |  |  |  | 1.21 | 1.09-1.36 | <0.001 |
| Race (ref = Black) |  |  |  |  |  | <0.001 |  |  | <0.001 |
| White |  |  |  | 0.79 | 0.68-0.92 |  | 0.77 | 0.66-0.89 |  |
| Other |  |  |  | 1.14 | 0.88-1.47 |  | 1.04 | 0.81-1.34 |  |
| Unknown |  |  |  | 0.74 | 0.18-3.11 |  | 0.64 | 0.15-2.68 |  |
| Admit from SNF |  |  |  | 0.60 | 0.45-0.78 | <0.001 |  |  |  |
| Congestive heart failure | 0.68 | 0.60-0.78 | <0.001 | 0.72 | 0.63-0.83 | <0.001 | 0.71 | 0.62-0.83 | <0.001 |
| Valvular disease |  |  |  |  |  |  | 0.87 | 0.70-1.07 | 0.18 |
| Pulmonary circulation disease |  |  |  |  |  |  | 0.82 | 0.66-1.01 | 0.06 |
| Peripheral vascular disease |  |  |  |  |  |  | 0.79 | 0.63-0.99 | 0.04 |
| Hypertension |  |  |  | 0.85 | 0.76-0.95 | 0.005 | 0.81 | 0.72-0.91 | <0.001 |
| Collagen vascular disease |  |  |  |  |  |  | 0.81 | 0.62-1.07 | 0.13 |
| Obesity | 0.68 | 0.58-0.81 | <0.001 | 0.71 | 0.60-0.85 | <0.001 | 0.70 | 0.59-0.84 | <0.001 |
| Alcohol abuse |  |  |  |  |  |  | 1.35 | 1.09-1.67 | 0.007 |
| Drug abuse | 1.66 | 1.35-2.04 | <0.001 | 1.57 | 1.27-1.93 | <0.001 |  |  |  |
| Diabetes |  |  |  | 0.83 | 0.73-0.94 | 0.004 | 0.83 | 0.73-0.94 | 0.004 |
| Immunosuppression |  |  |  |  |  |  | 0.95 | 0.82-1.11 | 0.53 |
| Dementia |  |  |  |  |  |  | 0.89 | 0.72-1.10 | 0.28 |
| Low functional status |  |  |  |  |  |  | 0.84 | 0.71-0.99 | 0.04 |
| Deficiency anemia |  |  |  |  |  |  | 0.92 | 0.81-1.05 | 0.2 |
| Pressure ulcer |  |  |  |  |  |  | 0.9 | 0.69-1.17 | 0.43 |
| Smoker | 1.26 | 1.12-1.43 | <0.001 | 1.23 | 1.09-1.39 | 0.001 | 1.27 | 1.11-1.45 | <0.001 |
| Chronic kidney disease |  |  |  |  |  |  | 1.20 | 1.02-1.40 | 0.03 |
| Stroke |  |  |  |  |  |  | 0.84 | 0.68-1.04 | 0.11 |
| Seizure/epilepsy |  |  |  |  |  |  | 0.93 | 0.71-1.20 | 0.57 |
| Thrombocytopenia |  |  |  |  |  |  | 1.23 | 1.01-1.49 | 0.046 |
| Hyponatremia | 1.34 | 1.17-1.54 | <0.001 | 1.35 | 1.17-1.55 | <0.001 | 1.34 | 1.16-1.54 | <0.001 |
| NIV on first 48 hours of admission |  |  |  |  |  |  | 1.1 | 0.93-1.31 | 0.28 |
| Urban |  |  |  |  |  |  | 0.97 | 0.75-1.27 | 0.84 |
| Teaching hospital |  |  |  | 0.85 | 0.76-0.95 | 0.004 | 0.87 | 0.78-0.98 | 0.02 |
| Regional area (ref = Midwest) |  |  |  |  |  | 0.001 |  |  | 0.003 |
| Northeast |  |  |  | 1.3 | 1.06-1.58 |  | 1.27 | 1.04-1.55 |  |
| South |  |  |  | 1.2 | 1.05-1.37 |  | 1.21 | 1.06-1.38 |  |
| West |  |  |  | 0.88 | 0.69-1.12 |  | 0.91 | 0.72-1.16 |  |
| IV antibiotics in past 1 year | 0.63 | 0.50-0.79 | <0.001 | 0.65 | 0.52-0.82 | <0.001 |  |  |  |
| Chronic lung disease |  |  |  |  |  |  | 1.11 | 0.99-1.24 | 0.08 |
| Chronic liver disease |  |  |  |  |  |  | 1.33 | 1.04-1.71 | 0.03 |
| Severe CAP on admission | 1.71 | 1.52-1.93 | <0.001 | 1.76 | 1.56-1.98 | <0.001 | 1.71 | 1.50-1.94 | <0.001 |
| SP infection in past year | 6.96 | 4.26-11.37 | <0.001 | 6.99 | 4.27-11.46 | <0.001 |  |  |  |

Abbreviations: AIC, Akaike information criterion; SNF, skilled nursing facility; NIV, non-invasive ventilation; SP, *Streptococcus pneumoniae*

**References:**

1. Riley LK, Rupert J. Evaluation of Patients with Leukocytosis. Am Fam Physician. 2015;92(11):1004–11.

2. Hoorn EJ, Zietse R. Diagnosis and Treatment of Hyponatremia: Compilation of the Guidelines. J Am Soc Nephrol JASN. 2017 May;28(5):1340–9.

3. Gauer RL. Thrombocytopenia. Am Fam Physician. 2012;85(6):612–22.

4. Centers for Disease Control and Prevention. Definitions of Symptoms for Reportable Illnesses. 2022. Available at https://www.cdc.gov/quarantine/air/reporting-deaths-illness/definitions-symptoms-reportable-illnesses.html#:~:text=CDC%20considers%20a%20person%20to,a%20history%20of%20feeling%20feverish.. Accessed 25 May 2022.
